# Modeling the Electric Potential across Neuronal Membranes: The Effect of Fixed Charges on Spinal Ganglion Neurons and Neuroblastoma Cells

**DOI:** 10.1371/journal.pone.0096194

**Published:** 2014-05-06

**Authors:** Thiago M. Pinto, Roseli S. Wedemann, Célia M. Cortez

**Affiliations:** 1 Instituto de Matemática e Estatística, Universidade do Estado do Rio de Janeiro, Rio de Janeiro, Brazil; 2 Departamento de Física, Faculdade de Filosofia, Ciências e Letras de Ribeirão Preto, Universidade de São Paulo, Ribeirão Preto, São Paulo, Brazil; University of Missouri, United States of America

## Abstract

We present a model for the electric potential profile across the membranes of neuronal cells. We considered the resting and action potential states, and analyzed the influence of fixed charges of the membrane on its electric potential, based on experimental values of membrane properties of the spinal ganglion neuron and the neuroblastoma cell. The spinal ganglion neuron represents a healthy neuron, and the neuroblastoma cell, which is tumorous, represents a pathological neuron. We numerically solved the non-linear Poisson-Boltzmann equation for the regions of the membrane model we have adopted, by considering the densities of charges dissolved in an electrolytic solution and fixed on both glycocalyx and cytoplasmic proteins. Our model predicts that there is a difference in the behavior of the electric potential profiles of the two types of cells, in response to changes in charge concentrations in the membrane. Our results also describe an insensitivity of the neuroblastoma cell membrane, as observed in some biological experiments. This electrical property may be responsible for the low pharmacological response of the neuroblastoma to certain chemotherapeutic treatments.

## Introduction

Electrostatic forces affect the passive and active transport of charged particles through biological membranes. The flow rate of ions through the membrane depends on the strength of the intramembranous electric field. These forces also affect the robustness of some ligands of the membrane [1]. In this work, we study the influence of surface electric charges on the stability of the cell membrane in the condition of equilibrium, by modeling the electric potential profile. The profile describes the behavior of the potential along the axis perpendicular to the cell membrane, from the outer bulk region to the inner cytoplasmic region [2]–[5]. We do not consider here dynamical phenomena in the structure of the membrane, and treat only the electrostatic situation, which occurs once the system has reached equilibrium. We refer the reader to studies such as [6], [7] that treat dynamical, nonequilibrium phenomena, like the molecular dynamics of ion channels associated with transmembrane ion transport, using the Poisson-Nersnt-Planck theory [6] and the Poisson-Boltzmann-Nernst-Planck model [7].

The electric potential on a cell surface is determined as the difference of potential between the membrane-solution interface and the bulk region [1]. It has been shown that the electrophoretic behavior of neuroblastoma cells provides information about their surface charges, in different phases of the cellular cycle [8]–[10]. These experiments show that membrane anionic groups are mainly responsible for the surface charges of murine neuroblastoma cells [10]. It is known that neuroblastoma cells, like all other cancerous cells, multiply quickly. Alterations of the dynamics of cellular multiplication compromise the synthesis and structure of components of the membrane, with possible degradation of these components, promoting deformations of the structure and composition of the plasma membrane [11].

We show a detailed and revised description of the model more briefly presented by Cortez and collaborators in [3]–[5], which was originally used to simulate the squid giant axon. This model is based on the statistical mechanical theory of electrolyte solutions and electric double layers [12]–[15]. We then present a study that applies this model in a novel way to the neurons of mammals (mice) [16], [17], in order to investigate the alterations of the electric potential and therefore, the capability of transmitting electric signals in the membrane of cancerous neurons. Here, the spinal ganglion neuron denotes a healthy neuron, and the neuroblastoma cell represents a tumorous neuron. With simulations of this model, we compare the effects of charges fixed onto the inner surface of the membrane and those associated with cytoplasmic proteins, on the electric potential on the surfaces of the membranes of both types of cells, considering both natural states of neurons, the resting and the action potential (AP) states. The AP state refers to the state of the neuron in which it has been stimulated enough, so that its physico-chemical conditions are such that the transmembrane potential reaches the maximum value of the AP. The temporal evolution of the transmembrane potential was not considered. We also calculated the potential profile across the membrane, including data from electrophoretic experiments in our model.

## Methods

Cortez and collaborators have proposed in [4] an axon membrane model, to study how charges fixed onto the inner surface of the membrane and those associated with cytoplasmic proteins influence the electric potential of the squid axon membrane. In their work, the effects of divalent ions were included, with a numerical solution of the model equations. In the present study, we apply this model to healthy and cancerous *mammalian* neurons, to understand electrical characteristics of the membranes of these cells. We present the formal derivation of the complete model here, since it was not shown in [3], [4] and because we have found mistakes in some of the equations presented in [4]. We also describe, in Section *Surface Potentials*, a method for calculating electric potentials on the interfaces of the neuronal membrane, which was not discussed in previous work. We thus present here a more detailed and revised description of the theory and mathematical model of the electric potential across neuronal membranes in equilibrium, which was originally discussed in [4].

**Figure 1:**
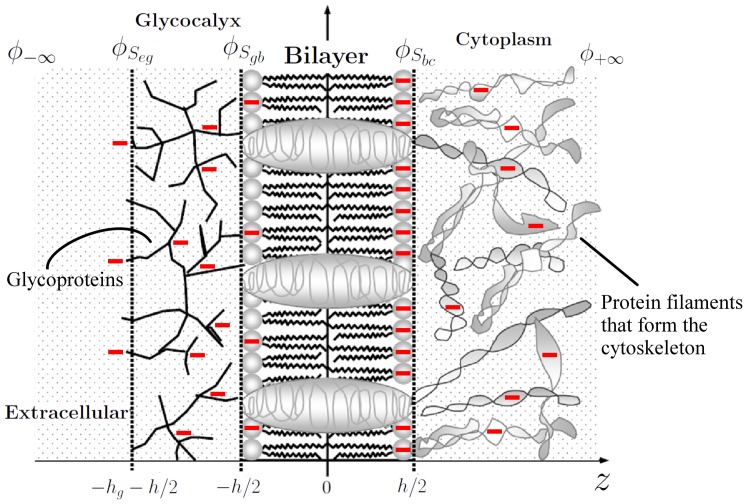
Model for a neuronal membrane. Different regions are presented, with the corresponding symbols for the potentials on the surfaces dividing regions. Symbols are explained in the text. Minus signs illustrate negative fixed charges on proteins.

In the neuronal membrane model we have adopted, shown in Fig. (1), four different regions are presented: extracellular, glycocalyx, bilayer and cytoplasm. The bilayer thickness is 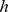 and the width of the glycocalyx is 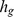. Surface potentials are represented as 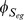 for the potential on the surface 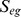, between the extracellular and glycocalyx regions, 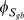 is the potential on the surface 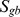, between the glycocalyx and the bilayer, and 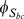 is the potential on the surface 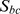, between the bilayer and cytoplasm. We denote by 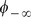 and 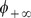 the potentials at 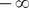, in the electrolytic extracellular phase, and at 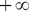, in the bulk cytoplasmic region, respectively.

### The Electric Potential in the Membrane Regions

To determine the potential profile across the membrane, we first consider the Poisson equation [3], [4], [15],
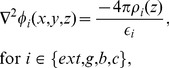
(1)where 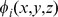 is the electric potential in any region 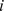; 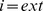 for the outer electrolytic region; 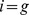 for the glycocalyx; 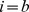 for the bilayer; and 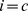 for the cytoplasm. The volumetric charge density due to the electrolytes in solution of area 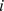 is 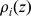, and 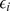 is the dielectric constant in region 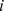. There is no charge in the bilayer (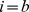), due to its hydrophobic property, and thus 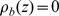.

We consider the further boundary conditions to calculate the constants of integration:

1. When 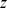 tends to an extreme value, 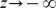 (in region 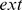) or 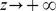 (in the cytoplasm), the electric potential tends to limiting values represented by 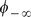 and 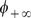, respectively. Ionic concentrations assume limiting values in the bulk regions, 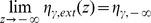 and 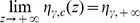, respectively, where 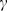 represents an ion, such as 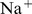, 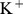, or 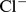.
2. When 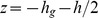 and 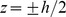, we consider the continuity of the electric potentials, 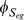, 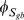 and 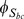, respectively.
3. There is a discontinuity of the electric field vector on the surfaces between regions.

#### The Effect of Fixed Charges

When we include the effect of fixed charges in the model, Poisson Eq. (1) becomes
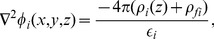
(2)where 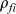 is the density of charges fixed onto proteins of area 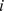 (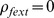, for the outer electrolytic region, 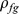 for the glycocalyx, and 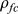 for the cytoplasm).

The volumetric charge density 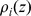 is the sum of the charge densities of positive and negative ions in the solution [3]

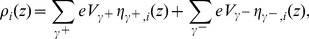
(3)where 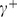 represents a positive ion, and 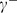, a negative ion. The molar density for an ion 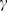 (ionic concentration) in region 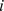 is 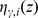, and 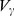 is the valency of ion 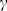. For example, 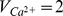 and 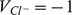. The absolute value of the electron charge is 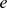. Due to the electroneutrality condition, we can write
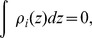
within the boundaries of each region.

In our model, we suppose that the surfaces are infinite in the 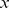 and 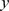 directions, perpendicular to 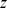, and that the distribution of charges in these directions is homogeneous. Considering this, and substituting Eq. (3) in Eq. (2), we obtain
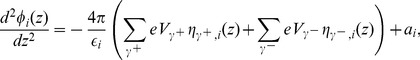
(4)where
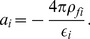
(5)To determine 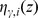, we use the equation for the electrochemical potential, due to an ionic solute in a diluted solution [3], [4]

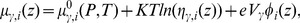
(6)where 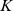 is Boltzmann's constant, 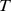 is the temperature, 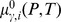 is the standard chemical potential, dependent on pressure and temperature, 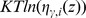 is a term that expresses the influence of the ionic concentration 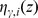, and 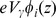 is the contribution of the electric potential.

Applying the 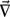 operator in Eq. (6) and again considering the homogeneous distribution of charges in the directions perpendicular to 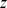, we verify that
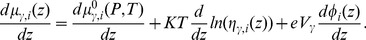
(7)Considering that there is a condition of Boltzmann equilibrium in the aqueous environments adjacent to the bilayer, 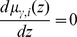 and 
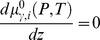
, and Eq. (7) becomes
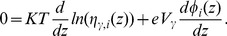
(8)The integration of Eq. (8) from 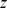 in one of the three regions, extracellular, glycocalyx and cytoplasm, to a limiting boundary region, for which we have experimentally measured quantities, considering the electrolytes distributed over the adjacency of the bilayer gives
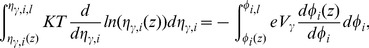
(9)where 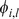 and 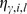 are limiting values of the electric potential and the ionic concentration of 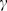, respectively, in region 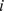. The solution of Eq. (9) results in
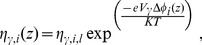
(10)where
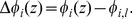
(11)The molar density for a positive 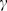 ion is thus given by
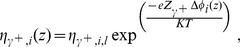
(12)and for a negative 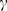 ion
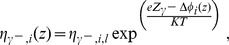
(13)where
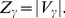
(14)
Equations (12) and (13) are the Boltzmann distribution of charges due to the presence of positive and negative 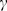 ions [15], respectively, in the phases adjacent to the bilayer. Substituting Eqs. (12) and (13) in Eq. (4), we obtain
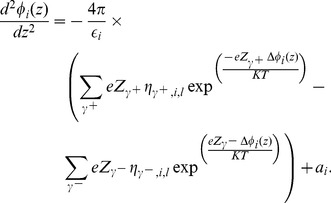
(15)In the bulk regions, we can consider the electroneutrality condition
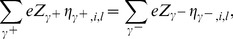
(16)and, in a first approximation, we assume a symmetric electrolyte to simplify our calculations, so that
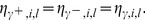
(17)We have taken the boundary values from experimental measurements in the bulk regions and on surface 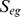, so that 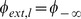, 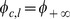, and 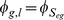. For the ionic concentrations, 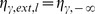, 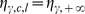, and 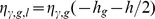. Throughout, we denote by 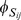 the electric potential on surface 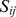 between regions 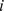 and 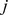.

We can now use Eq. (17) to rewrite Eq. (15) as
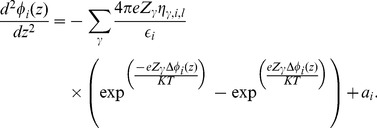
(18)If we consider that
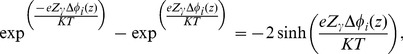
(19)we can write Eq. (18) as
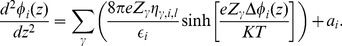
(20)Our model only considers mono (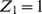) and divalent (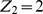) ions [4]. We thus limit the 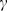 sum to
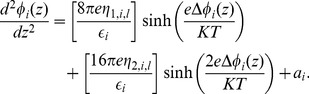
(21)To simplify further calculations, we denote
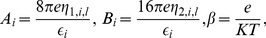
(22)and Eq. (21) may be expressed as
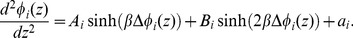
(23)Considering that
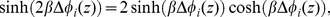
(24)Eq. (23) can be rewritten as
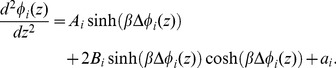
(25)Multiplying Eq. (25) by
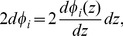
(26)and integrating, we have
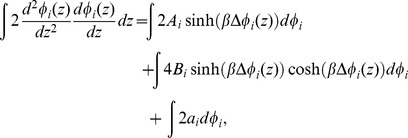
(27)whose solution is
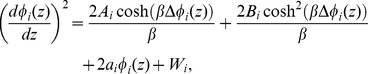
(28)where 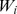 is a constant of integration for region 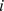.

To further simplify the symbolic representation of the equations, considering Eqs. (5) and (22), we denote
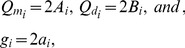
(29)where 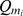 and 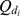 express the influence of boundary value concentrations of monovalent and divalent ions, respectively, in each region 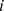. Eq. (28) is thus expressed as
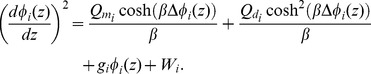
(30)We can rewrite Eq. (30) as
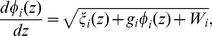
(31)where
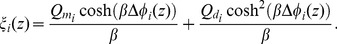
(32)
Equation (31) is the Poisson-Boltzmann equation for the electric potential in any region 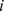.

#### Solution of the Poisson-Boltzmann Equation for the Extracellular Region


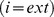


In the extracellular region, the effect of fixed charges is negligible (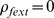 and 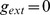), and the solution of Eq. (31) therefore only considers the electrolytic charges. Moreover, the electric potential in 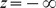 is constant and we can write
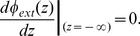
(33)In order to use Eq. (31) to calculate the potential in the extracellular region, we must find the value of 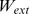. We thus consider an imaginary surface 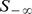, perpendicular to the 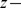
axis, at 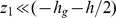, where 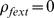. We then integrate Eq. (31) from another position 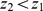 to 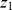. Since both 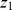 and 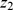 are in the 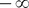 region, 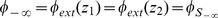, and 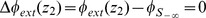. We can then substitute Eq. (33) and 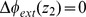 in Eq. (31), to calculate 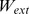 as
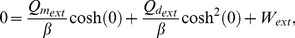
(34)and
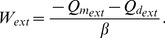
(35)We then substitute Eq. (35) in Eq. (31) to obtain the differential equation for 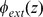, for any position 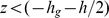, in the extracellular region
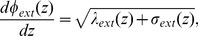
(36)where
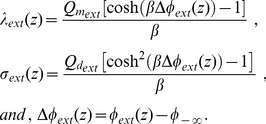
(37)
Equation (36) can be simplified to
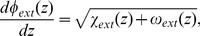
(38)where
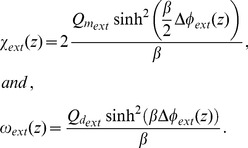
(39)


#### Solution of the Poisson-Boltzmann Equation for the Cytoplasmic Region


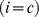
Because, in the cytoplasmic region, the potential in 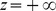 is also constant,
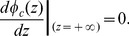
(40)As we did for the extracellular region, to calculate the potential in the cytoplasmic region using Eq. (31), we must first find the value of 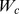. We thus consider an imaginary surface 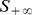, perpendicular to the 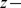
axis, at 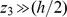. We then integrate Eq. (31) from another position 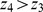 to 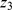. As both 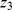 and 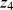 are in the 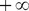 region, 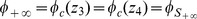, and 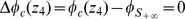. We thus substitute Eq. (40) and 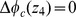 in Eq. (31), to obtain 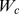 as
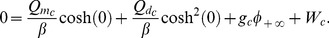
(41)The constant of integration 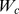 is
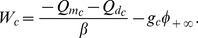
(42)We then substitute Eq. (42) in Eq. (31) to obtain the differential equation for 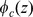, for any 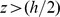, in the cytoplasmic region
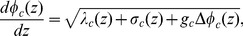
(43)where
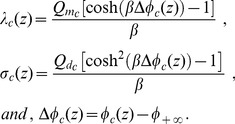
(44)
Equation (43) can be simplified to
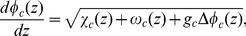
(45)where
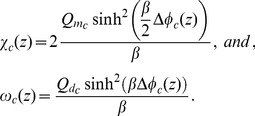
(46)


#### Solution of the Poisson-Boltzmann Equation for the Glycocalyx Region


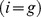
We consider that the potential 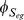 on the surface 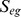 satisfies
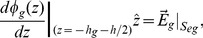
(47)where 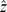 is the unit vector in the 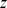 direction, and 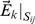 is the electric field in region 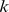, 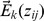, at 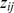, the position of surface 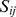 between regions 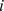 and 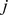, e.g., 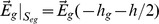 is the electric field in the glycocalyx region at the position of surface 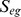.

We have previously stated that the boundary condition for ionic concentrations in the glycocalyx is defined as 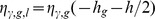. In order to obtain 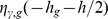 from the experimentally measured 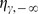, we would need to apply Eqs. (12) and (13). This would result in four values of 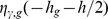 (for positive and negative, monovalent and divalent ions). In this case, the symmetric electrolytes assumption (Eq. (17)) would not hold, and the mathematical formalism leading to Eq. (31) would not apply. Nevertheless, in Eq. (15), the difference in the contributions of the terms involving 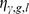 for the different ions is small compared to the value of the 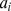 term, which is a few order of magnitude larger. We thus assume that 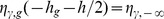, for monovalent and divalent ions.

In order to use Eq. (31) to calculate the potential in the glycocalyx region, we need to find the value of 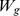. We thus solve Eq. (31) at 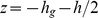, on the surface 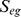, and take 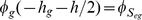, and 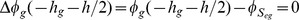. We can then substitute Eq. (47) and 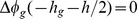 in Eq. (31), to calculate 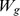 as
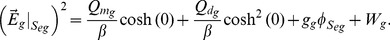
(48)The constant of integration 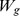, for the glycocalyx region, is therefore
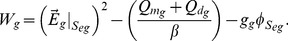
(49)We then substitute Eq. (49) in Eq. (31) to obtain the differential equation for 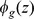, for any position 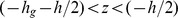, in the glycocalyx region
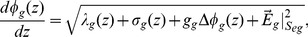
(50)where
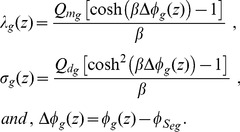
(51)
Equation (50) can be further simplified to
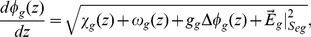
(52)where
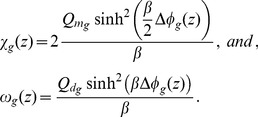
(53)


#### Solution of the Poisson-Boltzmann Equation for the Bilayer


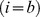
As mentioned earlier, because the bilayer is highly hydrophobic, 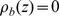, and therefore Eq. (2) assumes the form
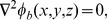
(54)and its solution is a family of linear functions. The electric field within the bilayer (see Eq. (59)), 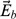, can thus be expressed as
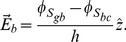
(55)


### Surface Potentials

In order to solve the differential Eqs. (38), (45) and (52) for the extracellular, cytoplasmic and glycocalyx regions of the neuronal membrane, respectively, we must know the values of the surface potentials 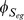, 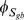 and 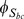. Although membrane surface potentials in cells cannot be measured experimentally, it is possible to obtain analytical predictions for the values of 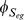, 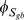 and 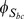, from basic electrostatic relations, as we will now show.

Considering the surface densities of electric charges, Gauss' law, and the discontinuity of the electric field vector on the surfaces 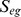, 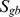 and 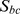, we obtain
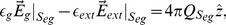
(56)




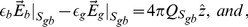
(57)




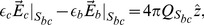
(58)respectively, where 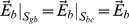 (see Eq. (55)). In the above, 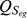, 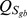 and 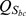 stand for the charge density on the 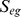, 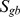 and 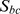 surfaces, respectively.

As
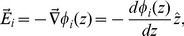
(59)in order to determine the discontinuity of the electric field vector on the surfaces 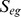, 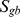 and 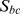, we substitute Eq. (59) in Eqs. (56), (57) and (58), and obtain
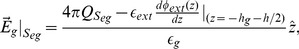
(60)




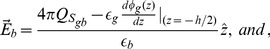
(61)




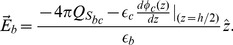
(62)As we have previously obtained the expression that determines the electric field within the bilayer, 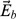, we substitute Eq. (55) into Eqs. (61) and (62), and obtain
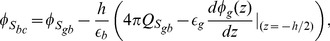
(63)




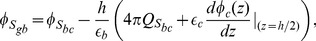
(64)respectively.

Substituting Eqs. (52) and (60) in Eq. (63), we determine the expression to calculate the surface potential 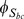


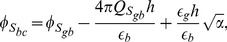
(65)where
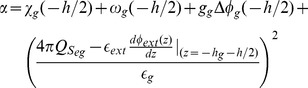
(66)and here, 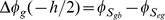. In the same manner, substituting Eq. (45) in Eq. (64), we determine the expression to calculate the surface potential 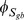


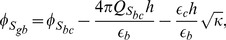
(67)where
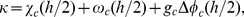
(68)and here, 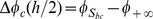.

The electric potential on the surface 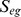, 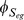, is determined from electrophoretic experiments [9], [16], [17] and the Helmholtz-Smoluchowski equation [3]

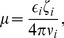
(69)where 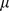 is the electrophoretic mobility, 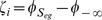 is the zeta potential, and 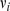 is the viscosity of region 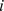. As, in our model, we define the potential 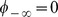, the surface potential 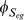 is
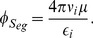
(70)


### Model Calculations for Spinal Ganglion Neurons and Neuroblastoma Cells

We have used data obtained from experimental observations [9], [16], [17] for values of parameters, in order to solve the first order ordinary differential equations, obtained from the Poisson-Boltzmann Eq. (31), for the different regions of the membrane. Some experimental values were obtained from electrophoretic experiments. Since each kind of cell presents a specific electrophoretic mobility, the values of some parameters are different for the spinal ganglion neuron and the neuroblastoma cell, in our calculations. Tables (1) and (2) show all experimental values of the parameters used to solve the equations for the ganglion and the neuroblastoma. The difference 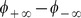 is called the transmembrane potential and is denoted as 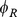 in the resting state, and 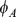 in the AP state. We have defined 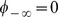 in our calculations, so that 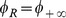 in the resting state, and 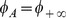 in the AP state. We have thus examined the influence of parameters that represent electrical properties of the membrane, on the resting and AP states, and analyzed the differences between the healthy spinal ganglion neuron and the neuroblastoma cell.

**Table 1:** Values of simulation parameters for both the spinal ganglion neuron and the neuroblastoma cell.

Parameter	Symbol	Value	Value in CGS	References
Dielectric constant in region 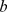	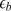	2	2	[3], [4]
Dielectric constant in region 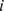 ( 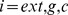 )	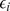	81	81	[4]
Glycocalyx width	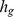	2.5 nm	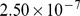 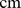	[4], [9], [16]
Bilayer thickness	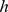	7.5 nm	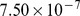 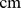	[3], [4], [9], [16]
Concentration of monovalent ions in bulk extracellular region	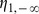	0.154 M	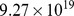 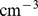	[16], [17], [29], [30]
Concentration of monovalent ions on 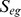	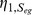	0.154 M	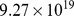 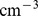	[16], [17], [29], [30]
Concentration of divalent ions in bulk extracellular region	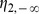	0.002 M	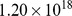 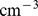	[16], [17], [29], [30]
Concentration of divalent ions on 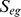	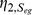	0.002 M	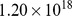 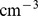	[16], [17], [29], [30]
Concentration of monovalent ions in bulk cytoplasmic region	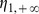	0.154 M	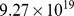 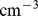	[29]–[31]
Concentration of divalent ions in bulk cytoplasmic region	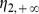	0.0004 M	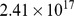 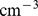	[29], [30]
Potential in 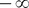 , the extracellular region	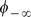	0 mV	0 statV	[3], [4]
Temperature	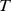	310 K	310 K	[4]
Boltzmann's constant	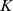	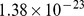 J/K	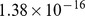 erg/K	[32]
Absolute value of electron charge	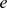	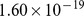 C	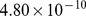 statC	[32]
Viscosity of region 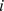	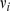	0.1 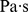	1 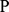	[3]

1 CGS is the centimeter-gram-second system of units.

**Table 2:** Parameter values specific to the spinal ganglion neuron (left) and to the neuroblastoma cell (right).

Parameter	Symbol	Spinal Ganglion Neuron			Neuroblastoma		
		Value	Value in CGS	Reference	Value	Value in CGS	Reference
Fixed charge density in glycocalyx	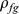	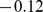 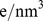	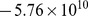 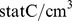	[16]	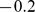 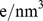	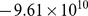 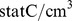	[9]
Charge density on 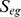	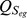	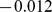 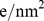	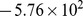 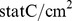	[16]	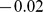 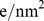	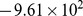 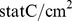	[9]
Charge density on 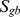	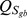	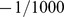 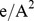	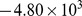 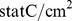	[33]	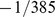 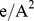	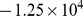 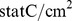	[33]
Electric potential on 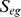	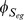	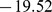 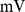	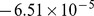 statV	Eq. (70)	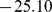 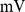	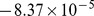 statV	Eq. (70)
Resting transmembrane potential	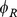	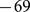 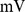	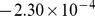 statV	[16], [34]	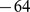 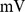	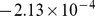 statV	[10]
Action transmembrane potential	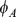	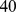 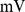	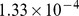 statV	[35]	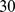 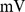	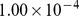 statV	[36]
Electrophoretic mobility	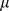	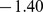 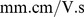	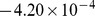 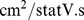	[9], [16]	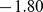 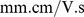	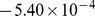 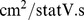	[9]

We implemented an approximate heuristic for finding roots of functions, to calculate 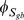 and 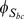 from Eqs. (65) and (67), which is specified in the next subsection. As mentioned earlier, the potential 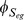 was calculated with Eq. (70), from data obtained from electrophoretic experiments.

As we included the density of charges fixed onto proteins within the membrane regions in the Poisson Eq. (1), we obtained a non-linear Poisson-Boltzmann Eq. (31), whose analytical solution has not been found. We therefore calculated values of the potential profiles with Eqs. (38), (45) and (52) numerically, using the Runge-Kutta method. The model simulation code is available on GitHub at https://github.com/pintotm/PLoSOne2014.

#### Roots of the System of Non-linear Equations

Because there is no experimental method to directly measure values of the surface potentials 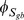 and 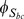, we use Eqs. (65) and (67) that form a system of non-linear equations with two variables, 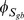 and 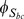, to determine these values. Some research work [3], [4], [9], [16], [17] indicates that the values of these potentials in real cells are in a limited region of the 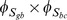 plane. This means that we are looking for one of the roots of Eqs. (65) and (67), in a known region.

We can write the system of Eqs. (65) and (67) as
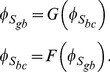
(71)Our simple method for finding the roots of this system consists in the steps described in *Algorithm_Roots*. At the resting state, 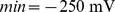 and 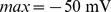, while, at the AP state, 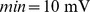 and 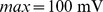.

#### Algorithm

*Algorithm Roots*

Step 0: 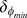  = 10^−4^ mV

Step 1: Choose an initial value for 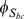


in min < 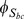 < max

Step 2: 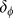  = 20 mV

Step 3: While 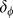 > 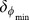 do

**begin**


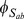
  =  G(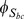)


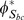
 =  F(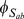)

**If** 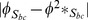 < 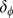 **then**


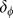
  =  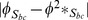



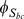
 =  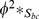


**else**

return to Step 1

**end if**

**end While**

#### Algorithm for determining 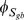 and 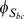, for the spinal ganglion neuron and the neuroblastoma cell

With the 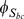 value found with this procedure, we use Eq. (67) to obtain the corresponding 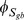 value. The surface potentials 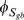 and 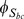 for the spinal ganglion neuron and the neuroblastoma cell, whose values are shown in the following section, were obtained with *Algorithm_Roots*.

## Results

The hydrophilic heads of the phospholipids that form the surfaces of the bilayer are negatively charged or polarized, and attribute a fixed charge density to surfaces 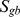 and 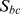. In the situation of electrostatic equilibrium, which we are analyzing, both bilayer surfaces, 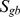 and 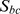, are surrounded by a “diffuse electric layer” formed by the motion of free ions in the fluid ionic solution, under the influence of electric attraction and thermal motion [12]–[15].

It is known that the inner surface charge density (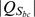) of the membrane is significantly higher than the outer surface density, due to the presence of negatively charged heads of phospholipids on the inner surface (phosphatidylserine), while on the outer surface the presence of neutral phospholipids dominates [18]. Moreover, the net charges fixed onto cytoplasmic proteins (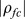) are considered to be higher than the fixed net charges distributed in the glycocalyx region [19]–[21].

With our mathematical model, we first investigate the effect of 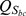 and 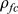 on the electric potential on the surfaces of the neuronal membranes of the spinal ganglion and the neuroblastoma. However, there is little information in the literature, regarding experimentally obtained quantities related to electric charges fixed within biological membranes. This is mainly due to the difficulties involved in obtaining the experimental measurements of these quantities. We therefore examine the behavior of the potentials 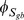 and 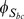 for a range of values of the ratios 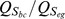 and 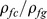, given known experimental values of 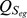 and 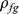, i.e. 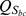 and 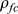 are multiples of 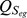 and 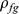, respectively. We note that both glycocalyx and cytoplasm and their surfaces are negatively charged, so that 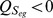, 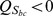, 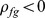 and 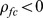.

**Figure 2:**
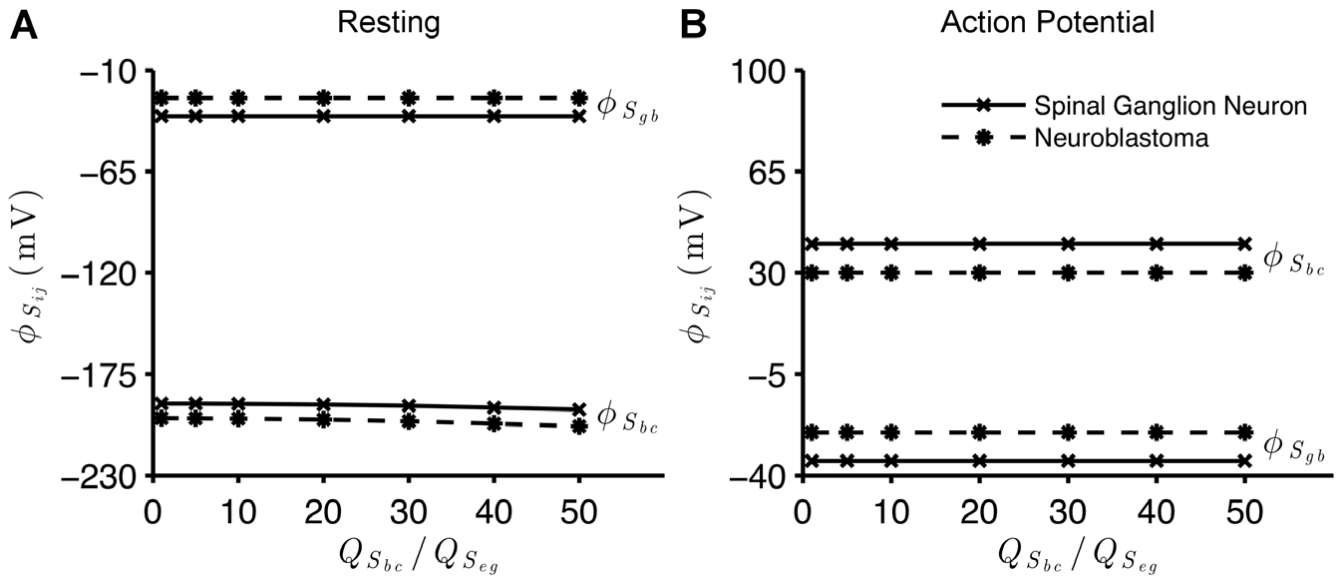
Sensitivity of the membrane surface potentials to inner surface charge density. Electric potential on the surfaces of regions of the membranes of the spinal ganglion neuron (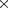) and the neuroblastoma cell (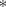), as a function of the ratio 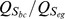, as 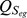 is kept constant. In the resting state (A), 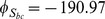 mV, for the ganglion neuron and 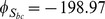 mV, for the neuroblastoma, when 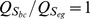 (maximum values), while 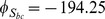 mV (ganglion) and 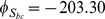 mV (neuroblastoma), for 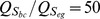 (minimum). In the AP state (B), 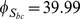 mV, for the ganglion neuron and 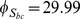 mV, for the neuroblastoma, when 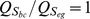 (maximum), while 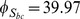 mV (ganglion) and 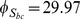 mV (neuroblastoma) for 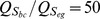 (minimum). In all simulations, for resting and AP states, 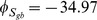 mV, for the ganglion, and 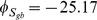 mV, for the neuroblastoma. In both graphs, 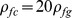.

Figure (2) shows the behavior of 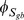 and 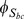 as we increase the negative charge on 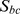, i.e. as we decrease 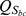. We notice that for both the resting and AP states, a decrease of 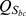 has almost no effect on the surface potentials of both neuronal membranes. These variations in 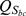 only determine a small gradual decay of 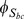 during the resting state of the cells. The resting and AP states were specified by boundary conditions, i.e. specific parametric values applied to the model. Values for 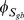 and 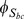 are different between these types of cells, due to their specific membrane properties. We also observe that 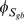 remains constant at 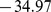 mV and 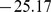 mV, respectively for the spinal ganglion and neuroblastoma cells, when they switch from the resting to AP states, and vice versa. Moreover, during the AP state, 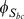 assumes values near the transmembrane potentials (Fig. (2B)).

**Figure 3:**
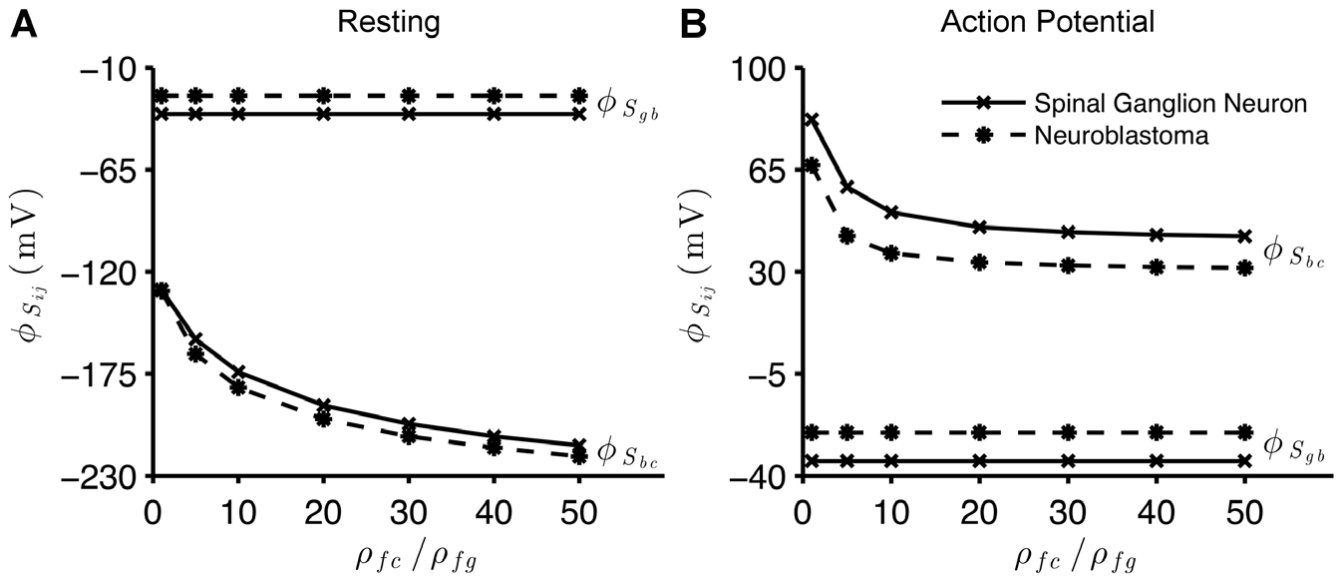
Sensitivity of the membrane surface potentials to charge density in the cytoplasm. Electric potentials 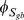 and 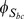 as a function of 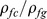, as 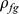 is kept constant, for the spinal ganglion neuron (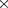) and the neuroblastoma cell (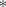). In the resting state (A), 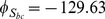 mV, for the ganglion neuron and 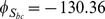 mV, for the neuroblastoma, when 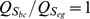 (maximum values), while 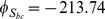 mV (ganglion) and 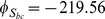 mV (neuroblastoma), for 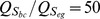 (minimum). In the AP state (B), 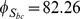 mV, for the ganglion neuron and 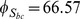 mV, for the neuroblastoma, when 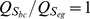 (maximum), while 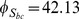 mV (ganglion) and 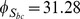 mV (neuroblastoma) for 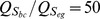 (minimum). In all simulations, for resting and AP states, 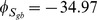 mV, for the ganglion, and 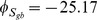 mV, for the neuroblastoma. In both graphs, 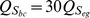.

We also examine the electric potential on the surfaces of the membranes of the healthy and the cancerous cells, in response to variations in the density of charges fixed onto proteins of the cytoplasm. Figure (3) presents the resulting 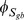 and 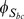 as we increase this density of negative charges in the cytoplasm, i.e. as we decrease 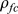. For both the resting and AP states, 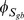 remains constant when values of 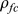 vary. However, a decrease in 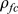 causes a substantial fall of 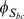, for both types of cells, at resting and AP states. At the AP state, 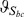 presents a quick drop when 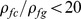 and tends to an asymptotic value, for decreasing values of 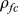, for both types of cells.

**Figure 4:**
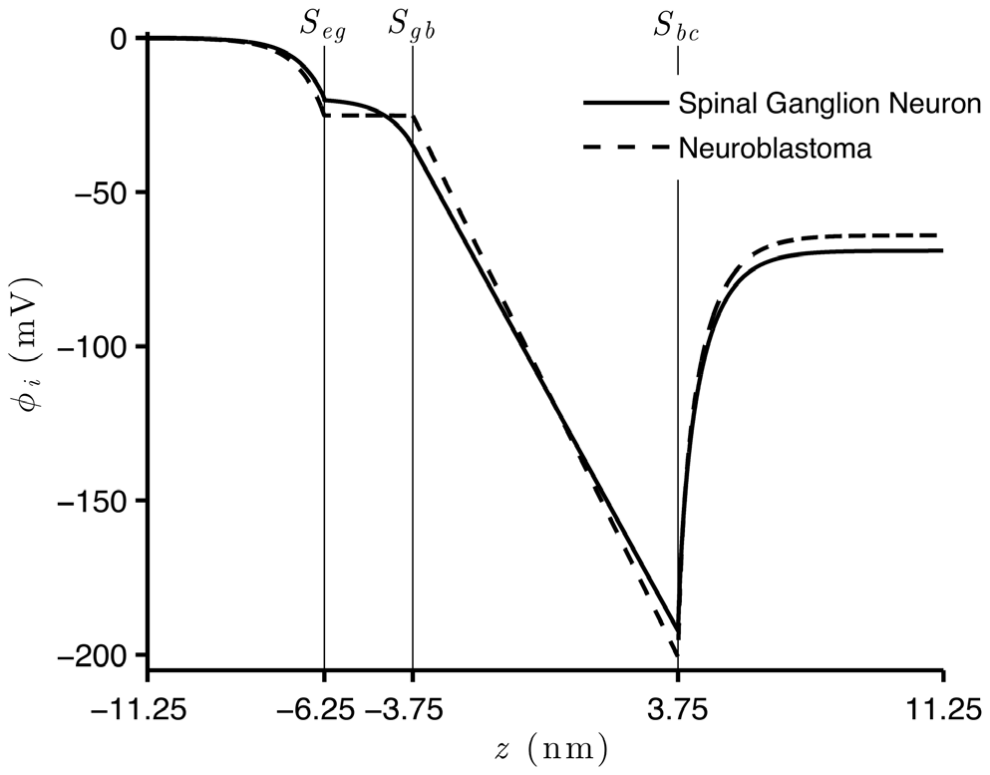
Electric potential across the membranes of spinal ganglion neurons and neuroblastoma cells, during resting state. Solutions of Eq. (52) with boundary 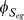, and Eq. (45) with boundary 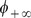 = 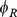 result respectively in 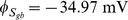 and 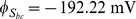, for the spinal ganglion neuron (solid), and for the neuroblastoma cell (dashed) in 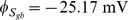 and 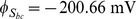. For all simulations, 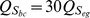 and 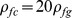.

**Figure 5:**
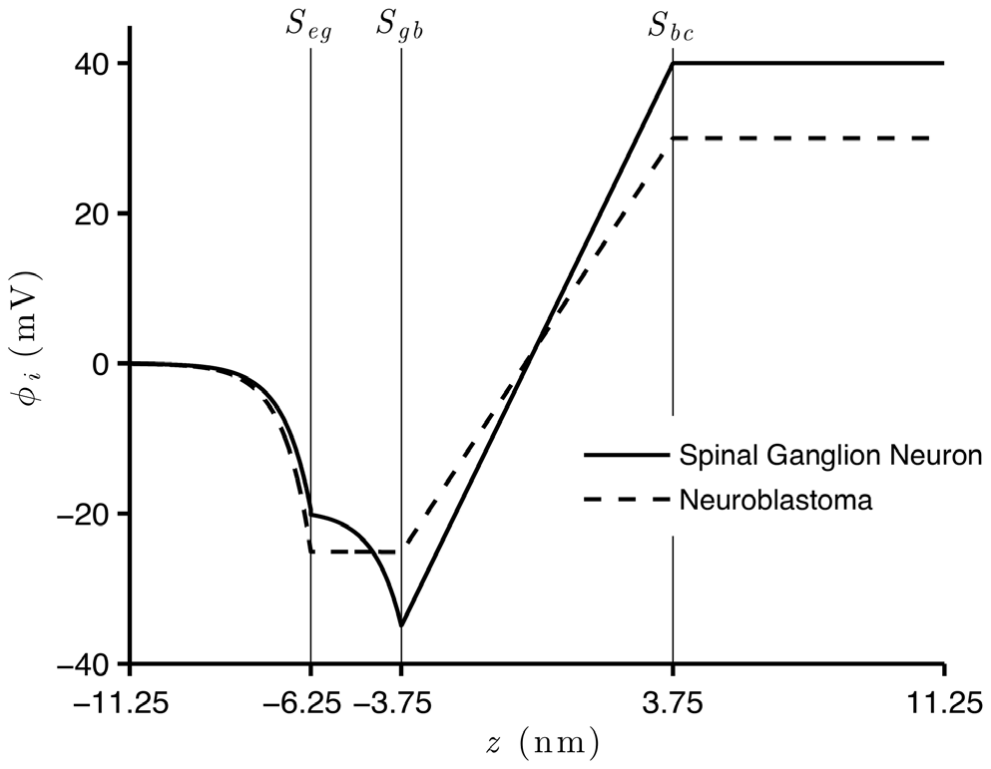
Electric potential across the membranes of spinal ganglion neurons and neuroblastoma cells, during AP state. Solutions of Eq. (52) with boundary 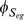, and Eq. (45) with boundary 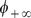 = 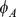 result respectively in 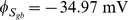 and 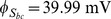, for the spinal ganglion neuron (solid), and for the neuroblastoma cell (dashed) in 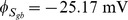 and 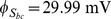. For all simulations, 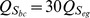 and 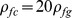.

Besides investigating the effect of fixed charges on the potential on the surfaces of these membranes, we study how the electric potential profile changes across the membranes of spinal ganglion neurons and neuroblastoma cells, for the resting and AP states. Although the values of 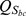 and 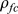 are not known, 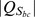 and 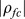 are much larger than the corresponding charges in the outer regions. We thus chose fixed values of 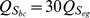 and 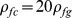 (which are the same fixed values in Figs. (3) and (2), respectively) to calculate the potential profile in Fig. (4), for the resting state and in Fig. (5), during the AP state. For both natural states of these cells, we verify an accentuated decrease of the potential along the 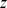 axis, from the extracellular region to the surface of the glycocalyx. This decay is slightly more substantial for the neuroblastoma than for the spinal ganglion neuron, although the shapes of both curves are very similar.

In the spinal ganglion neuron, the potential maintains a substantial drop across the glycocalyx. Interestingly, this phenomenon does not occur for the cancerous cells, whose electric potential remains almost unchanged in this region. We observe linear variations of the potential across the bilayer of these cells, for both resting and AP states. During rest, the electric potential assumes much lower values on 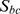 than the asymptotic value in the bulk cytoplasmic region, while during the AP state, these values are similar. Before reaching the transmembrane potential value, the intracellular potential exponentially increases from the inner surface of the membrane to the bulk cytoplasmic region, during the resting condition, while during the AP state, we see no alteration in the electric potential in the cytoplasm, for both cells.

## Discussion

There are other membrane phenomena due to electric charge effects that we have not considered in our model, such as the electric pump current [22]. We have not included these phenomena here as they seem to be negligible, in a first approximation, to the determination of the electric surface potentials. For example, Jäckle [22] states that “the net electric pump current is not a relevant parameter in the casual theory of the resting potential”.

Experiments have revealed important discrepancies in the electrical properties of spinal ganglion neurons and neuroblastoma cells [9], [16]. These differences are reflected in the parameter values we used in our simulations, as shown in Table (2). We notice that the values for the electrophoretic mobility, and the charge densities in the glycocalyx and on 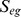 are much more negative for the neuroblastoma than for the ganglion. Because neuroblastoma cells contain a higher amount of negative 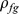, 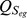 and 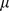 compared with ganglion neurons, we indeed expected to obtain lower 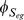 and 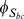 values for the neuroblastoma in our simulations.

Our simulations also demonstrate that variations in the electric charges fixed onto the inner surface of the membrane have a small effect on the electric potential of the surfaces that compose the neuronal membranes (Fig. (2)). We observe only a gentle gradual drop in 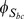 of the spinal ganglion and the neuroblastoma cells during the resting state, as charges fixed on 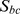 decrease (more negative values). However, our model shows that decreasing the density of charges fixed onto proteins of the cytoplasm (increasing 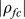 and 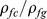) results in a substantial decay of 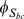, in both cells (Fig. (3)).

Nonetheless, variations of intracellular charges fixed on the membrane and on the cytoplasmic proteins have no effect on the potential on 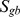 (Figs. (2) and (3)). This is related to the fact that the membrane plays a role in electrically isolating the intracellular and extracellular regions, due to the absence of charges within the lipidic bilayer (see Eq. (54)).

The results we obtained for the spinal ganglion neurons and the neuroblastoma cells are generally similar to those obtained for the squid axon membrane in [4]. These authors showed that a decrease of 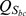 provokes a gentle decrease of 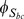 of the squid axon membrane. This behavior was also observed in our simulations for the membranes of spinal ganglion neurons and neuroblastoma cells. Nevertheless, their results indicate that a decrease of 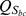 causes a sensitive increase of 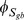 during the AP state and a small decrease of 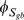 during the resting state, whereas our results show a constant 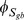 value for ganglion and neuroblastoma cells. The insensitivity of 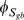 to variations of 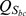 which we have found seems more reasonable, given the above mentioned isolating effect of the lipidic bilayer.

Cortez and collaborators [4] have shown that a decrease of 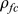 (in the same range of 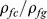 which we studied) causes practically no change in the surface potentials. A possible reason for this may be that the 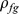 value for the squid axon is approximately zero, so that the values of 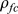, in the domain of their graphs, are very close to zero. In contrast, our simulations indicate that a decrease of 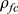 provokes an expressive fall of 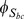. In our case, 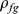 (and 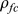) values for ganglion neurons and neuroblastoma cells are much more negative than those observed for squid axons and, therefore, a decrease of 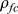 has a high influence on 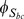.

An interesting result of our calculations is that, in the spinal ganglion neuron, the electric potential across the glycocalyx decreases, and this does not occur in the neuroblastoma cell. This reveals an important discrepancy of the electric fields in the glycocalyx of both types of cells (Figs. (4) and (5)), and may explain the difference between their electrophoretic behavior, which was observed in experiments [9], [16]. As expected, the electric potential presents a linear behavior within the bilayer of the membrane during the resting and AP states, due to the absence of electric charges in this region.

The strong negative electric potential on 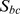 is a characteristic of the potential profile in the resting state, and this probably occurs for all types of neuronal cells (Fig. (4)). The steep increase of the potential from 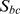 towards the bulk cytoplasmic region is regulated by the negative charges spatially distributed in the cytoplasm. Even though the net value of charges of proteins is predominantly negative in the cytoplasm, our simulations indicate that the contribution of these charges to the intracellular potential profile is much smaller than the effect of charges fixed on 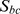. This is shown by the curvature of the potential in the cytoplasmic region.

The neuroblastoma cells, like all cancerous cells, multiply quickly. Alterations of the dynamics of cellular multiplication mediate changes in the synthesis, structure and degradation of the membrane components [11], which result in deformations on the structure and composition of the surfaces of membranes [23]. These deformations provoke changes in the composition of electric charges on the membrane. Our results indicate that the alteration of these charges and of those within the cells may influence the behavior of the potential on the inner surface of the neuroblastoma cells.

Experimental observations have suggested that the resting state and the generation of action potentials in human neuroblastoma cells depend on the degree of the morphological differentiation of the cell. Some of these cells are relatively non-excitable [24], [25]. Kuramoto et al. [26] stimulated the growth of SK-N-SH human neuroblastoma cells under standard culture conditions. These cancerous cells remained morphologically undifferentiated, partially responded to injections of pulses of electric current, and presented deficiency of the depolarizing component of the mechanism that generates the action potential. We included these findings in our simulations, and Fig. (5) is consistent with the fact that the depolarization of the electric potential in the neuroblastoma, during generation of the action potential, is less intense than in the healthy spinal ganglion neuron. The neuroblastoma should thus generate a lower firing rate in response to its input excitation, and this may affect the transmission of signals through networks of these neurons and their functions of storage and communication of information.

Mironov and Dolgaya [17] have suggested that the outer electric charges for the neuroblastoma cells and erythrocytes are similar, but the spinal ganglion neurons strongly differ from these cells. Therefore, the molecular structure (and the resulting constitution of charges) on the outer surface of the membrane of the neuroblastoma cells would be similar to the erythrocytes, and may be constituted by 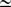 40% of peripheral proteins and 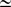 60% of gangliosides. Our results illustrate that the drop of the potential across the glycocalyx for the neuroblastoma cell is much smaller than for the spinal ganglion neuron, during both resting and AP states. This corroborates previous studies which show a smaller decay of the potential for the erythrocyte in the glycocalyx than for the neuron [2], [4], [5]. The different behavior of the potential across the glycocalyx, for the neuroblastoma and the spinal ganglion neuron, should indicate important differences among these cells, of the properties that enable the transmission of electric signals through the membrane. This occurs due to the fact that different molecular structures of these membranes interact differently with (i) the outer electric field, which is responsible for the orientation of the charged particles that are closer to the membrane, and (ii) the potential on the outer surface of the membrane. The nature of these interactions are crucial for many cell processes, such as the beginning of the process of triggering of the action potential, which depends on the opening of specific 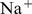 channels.

Our results may also contribute to understanding the resistance of the neuroblastoma to certain chemotherapeutic treatments [27], [28]. The smaller change of the potential, in response to changes in properties of cellular cultures (pH values, for instance) and to the amount of fixed charges present in the membrane due to alterations in its composition and structure, may be an electric property responsible for the low pharmacological response.
